# Metagenomic and proteomic analysis of bacterial retting community and proteome profile in the degumming process of kenaf bast

**DOI:** 10.1186/s12870-022-03890-5

**Published:** 2022-11-05

**Authors:** Huan Xu, Lixia Zhang, Xiangyuan Feng, Qi Yang, Ke Zheng, Shengwen Duan, Lifeng Cheng

**Affiliations:** 1 Institute of Bast Fiber Crops, Chinese Academy of Agricultural Science, 348 West XianJiahu Road, Changsha, China; 2 Xinyang City Academy of Agricultural Sciences, 20 Minquan South Street, Shihe District, Xinyang, Henan China

**Keywords:** Kenaf bast, Bacterial retting community, Metagenomic, Proteomic analysis, Proteome profile

## Abstract

**Background:**

Data on the microbial community and functional proteins associated with degumming in kenaf remains scant. Here, we analyzed the microbial communities associated with kenaf (*Hibiscus cannabinus*) bast fibers during retting to identify potential candidate degumming bacteria. Retting liquids were collected and analyzed at 0 days, 10 days, and 34 days and then evaluated the yield and quality of kenaf fiber at the different retting times. Besides, the microbial communities were characterized using metagenomic and proteomic analysis by LC–MS/MS technology.

**Results:**

The data showed that increase in the retting time significantly improves the softness, dispersion, and fiber whiteness of the kenaf fiber. The relative abundance of *Acinetobacter* increased from 2.88% at the baseline to 6.64% at the 34th retting. On the other hand, some members of *Clostridium* were reduced from 3% at the baseline to 2% at the 34th retting. Analysis of carbohydrate active enzymes showed constant changes in the utilization of carbohydrates. Besides, benzoquinone reductase, cellobiose dehydrogenase, glucose 1-oxidase, aryl alcohol oxidase and alcohol oxidase were the top five most abundant enzymes in the retting liquids. This present results demonstrated that the expressions of B7GYR8, Q6RYW5 and Q6FFK2 proteins were suppressed in *Acinetobacter* with the retting time. On the contrary, P05149 was upregulated with the retting time. In *Clostridium*, P37698, P52040 and P54937 proteins were upregulated with the retting time.

**Conclusion:**

In addition, bacteria *Acinetobacter* and *Clostridium* might be playing important roles in the kenaf degumming process. Similarly, up-regulation of P37698, P52040 and P54937 proteins is an important manifestation and mediates important roles in the degumming process.

**Supplementary Information:**

The online version contains supplementary material available at 10.1186/s12870-022-03890-5.

## Highlights

The growth of *Prevotella* was initially inhibited with the kenaf retting.

*Acinetobacter* can promote the degumming process of kenaf.

The upregulation of P37698, P52040 and P54937 proteins is a sign in the degumming.

**Supplementary Information:**

The online version contains supplementary material available at 10.1186/s12870-022-03890-5.

## Background

Kenaf (*Hibiscus cannabinus*), widely planted in India, China and Bangladesh, is an annual herbaceous bast fiber crop of the genus Malvaceae [1]. Except for a high content of 53–66% cellulose, kenaf contains 8–16% lignin, 23–35% pectin and hemicellulose [2–4]. These non-cellulose substances are called “gum”. To develop high-quality textile products, degumming is necessary for kenaf to eliminate the gum. Generally, kenaf fiber is obtained through field-retting in the south rural areas of China. However, retting is a major problem limiting large-scale application of kenaf in textile industry. Despite decades of research, little is known about the microbial communities involved in the degumming process. Previous studies focused on culturable isolates such as *Bacillus, Clostridium*, and *Pseudomonas* spp in the degumming process [2]. Recent studies have identified dominant bacteria in the degumming ecosystem, including *Clostridia, Pseudomonas* and *Bacteria* [3–5]. Some bacteria especially members of genera *Bacillus, Paenibacillus*, and *Clostridium* have been shown to produce pectinase [6]. The presence of these bacteria accelerated the degumming process. A previous study showed that pectinase and mannanase are key enzymes in the degumming of kenaf, which was mediated by bacteria, such as *Acinetobacter, Clostridium or Brevibacillus* [7]. Therefore, identification of microorganisms related to non-cellulolytic enzyme (such as pectinase, mannanase, xylanase, ligninolytic enzymes and so on) could have important contribution to the kenaf degumming process.

Metagenomic approach with direct sequencing of microbial genomes from environmental samples is a culture-independent method that enables identification of uncultured microbes [8]. Through metagenomics, the taxonomy and abundance of microorganisms in retting liquid samples can be detected. At the same time, it is possible to analyze the abundance of specific genes, especially those related to pectin-, hemicellulose-, lignin-degrading enzymes. Besides, the emergence of high throughput proteomics and metabolomics tools using LC/MS helps in the generation of large-scale data sets which can be used in the identification of microbial protein markers. Unlike metagenomics, which measures the functional potential of the microbial community, metaproteomic signatures provide a direct measure of the microbial activity of the community [9]. Due to the diversity of the kenaf gum and the complex mechanisms of enzyme-catalyzed degradation of non-cellulose, it is difficult to screen high-efficiency kenaf degumming strains. To date, the microbial community, and functional proteins of kenaf degumming remain unclear. Besides, there is lack of theoretical basis for screening and genetic construction of functional microorganisms.

In this study, retting liquid samples were collected at different times. Metagenomics and proteomic analysis were performed to analyze the microbial diversity and changes of functional proteins during the degumming process. Besides, we screened for dominant microorganisms and differentially expressed proteins of the degumming microorganisms in kenaf. This present study may provide a reference for screening of kenaf degumming microorganisms and lay the basis for further studies on the catalytic mechanisms of biological degumming, which will further help in improving the retting efficiency, and hence the fiber quality.

## Results

### The physical and chemical characteristics of the retting liquid

**Fig. 1:**
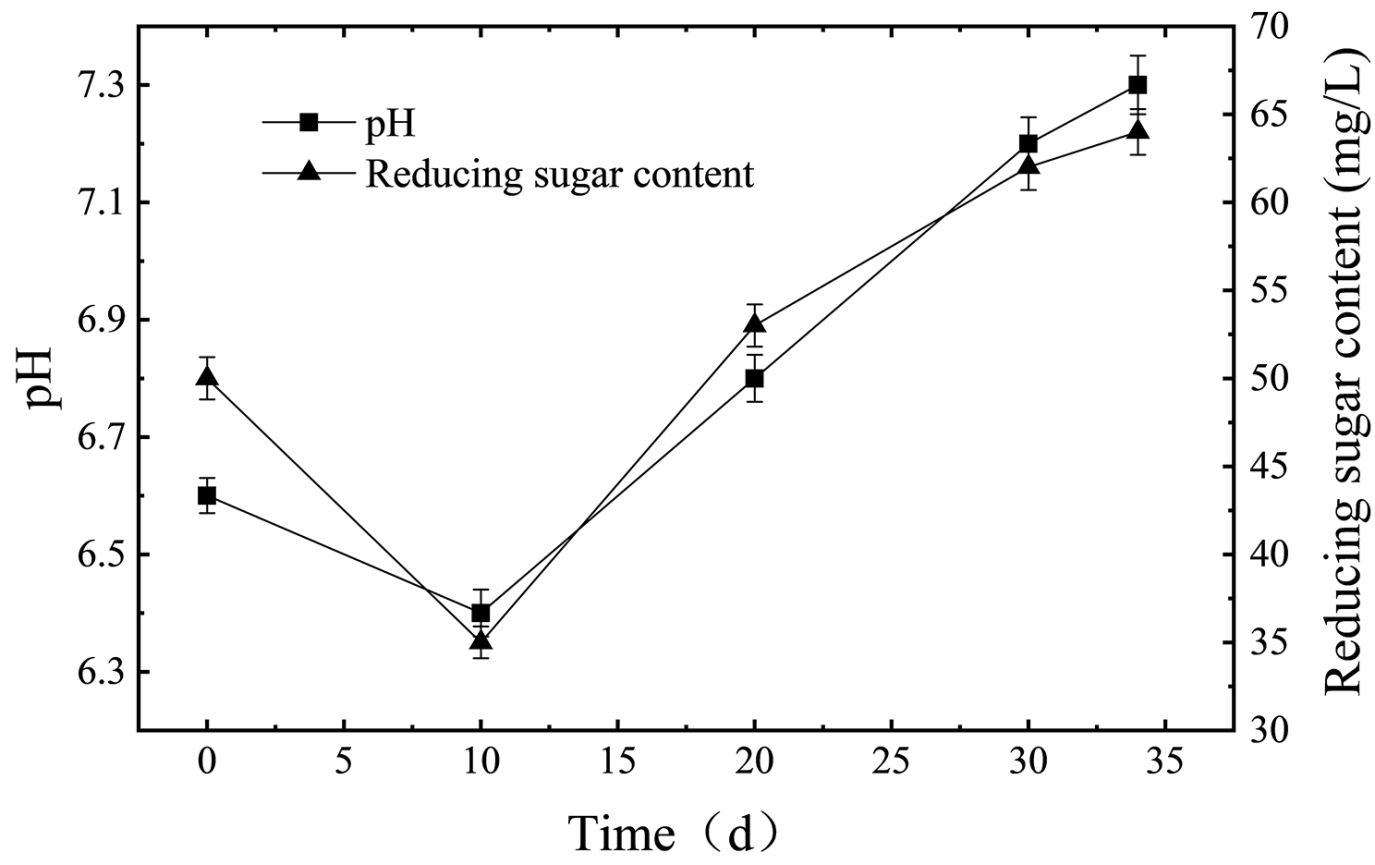
Changes of pH value and reducing sugar content in kenaf retting solution. The left-hand ordinate indicates the pH value and the right-hand ordinate indicates reducing sugar content in kenaf retting solution

The pH values of the retting liquid and reducing sugar characterize the biological fermentation environment in the retting process. A pH value is an important factor that affects microbial growth and degumming enzyme activity, generally, the ideal pH range is between 6.5 and 8.0. With the extension of retting time, the pH value of the retting solution decreased gradually, and reached the lowest value (pH 6.4) after 10 days; Thereafter, the pH increased continuously until retting for 34 days, with a pH of 7.3 (Fig. 1). Like the pH trend, the content of the reducing sugar in the kenaf retting liquid decreased continuously in the early stage of retting (the first 10 days) and then increased (Fig. 1). In summary, in the retting process, the trend of change of pH and reducing sugar content showed a “V” shape.

### Kenaf fiber morphology and yield

**Fig. 2:**
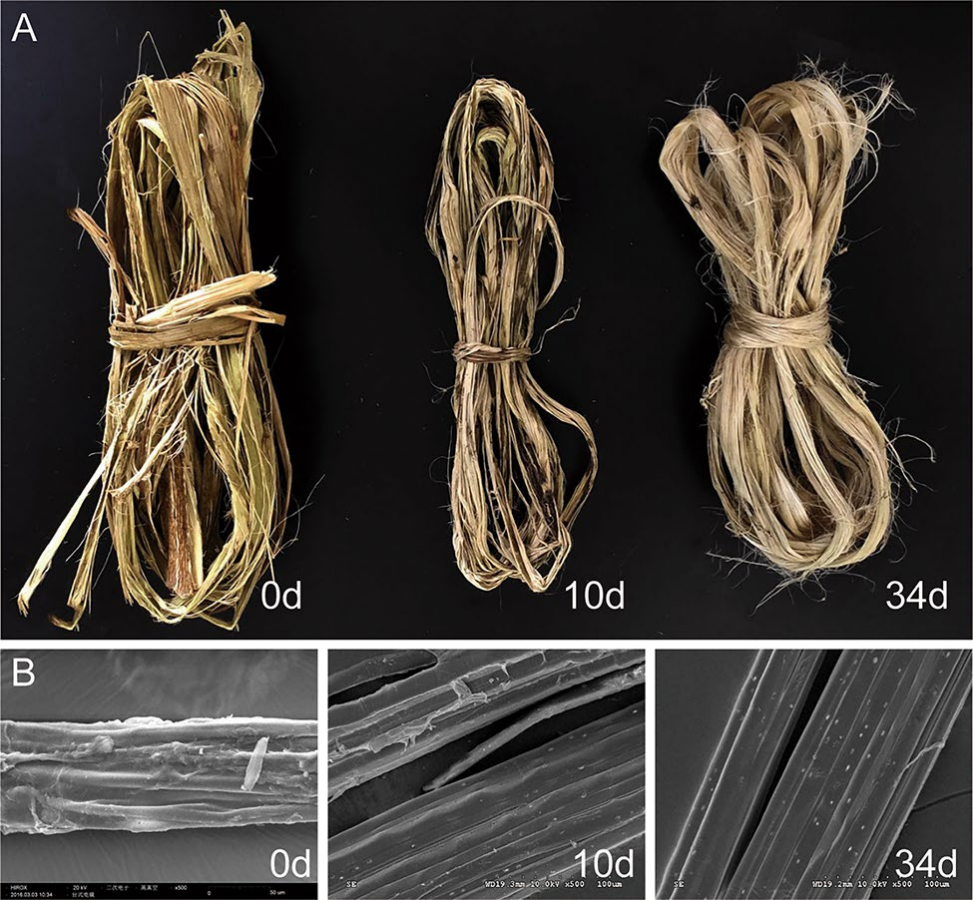
The appearance morphology and the fiber morphology scanned by electron microscope of kenaf at different retting times. (a) The appearance morphology of kenaf at different retting time. (b) The fiber morphology scanned by electron microscope (500×) of kenaf at different retting times

The qualitative analysis of the appearance of kenaf at different retting times was carried out. The data showed that as the retting time increases, the softness, dispersion and fiber whiteness of the kenaf fiber significantly improved (Fig. 2a; Table 1), which showed that increase in the retting time enhanced the retting effect. Scanning electron microscopy (SEM) can clearly assess the fiber surface morphology. The SEM results showed that, compared with the kenaf raw material, the kenaf fibers were partially degraded and begin to disperse after 10 days of retting. After 34 days, the fibers were completely dispersed, and the surfaces were smoother (Fig. 2b). The kenaf fiber yield was 63.9%.

**Table 1:** Morphology of Kenaf at different retting times

	0d	10d	20d	30d	34d
Softness	0	++	+++	++++	+++++
Dispersion	0	++	+++	++++	+++++
Color and lustre	0	++	+++	++++	++++
Comprehensive evaluation	0	++	+++	++++	+++++

Note: more “+” indicates that the softness, dispersion, color and lustre and comprehensive evaluation of the kenaf fiber is better

### Metagenomic sequence data

The quality control analysis of the raw sequencing data showed that the percentage of clean data was > 97% in the 12 samples with an average Q30 value of 94.92%, indicating that the sequencing data was of high quality (Table S1). The average length of assembled contigs was 968 bp with N50 and N90 value of 970 and 552, respectively (Table S1). Besides, the quality of the sequencing data and the assembled contigs met the standards for further analysis.

### Taxonomic classification

**Fig. 3:**
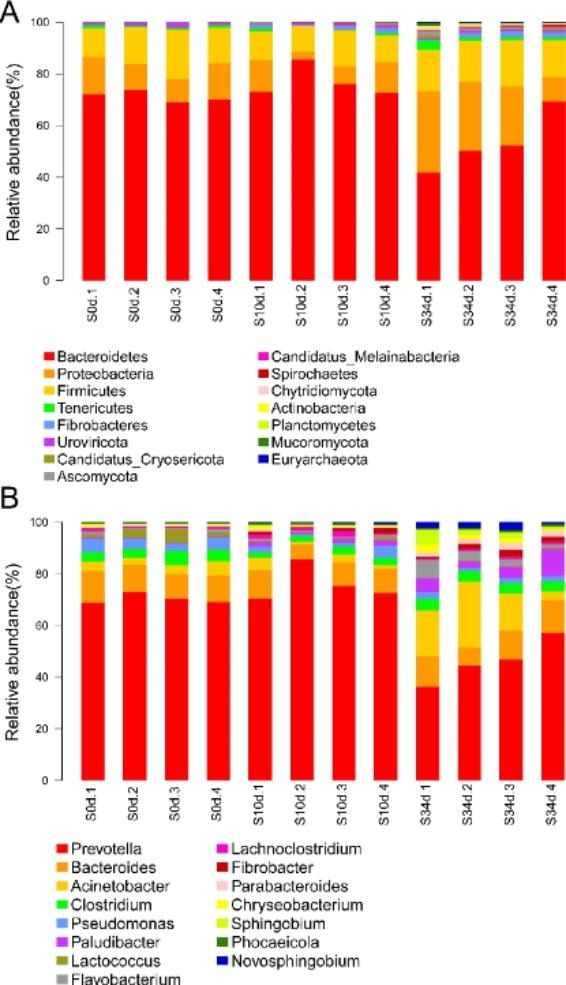
Relative abundance of bacteria in all retting liquid samples. Relative abundances at (A) phylum level and (B) genus level. The relative taxonomic abundances were calculated from metagenomic data. Taxonomy was assigned when reads showed ≥ 70% identity against subjects in the nucleotide (nt) database (NCBI). “S0d to S34d” refers to the type of samples (0d = baseline; 10 = retting samples for 10 days; 34d = retting samples for 34 days), the followed numbers refers to replicate samples

Taxonomic classification of raw sequences was assigned to 185 phyla, 170 classes, 352 orders, 795 families, and 3407 genera. The major phyla present in the retting liquid samples constituted more than 90% of the microbiota. At the phylum level, the most abundant phyla were Bacteroidetes, Proteobacteria, and Firmicutes. There was no significant difference in the relative abundance of the Bacteroidetes between the baseline and early stage of retting. However, the abundance of Bacteroidetes was greatly reduced at the end of retting (Fig. 3a). On the contrary, the abundance of Proteobacteria increased with the extension of the retting time (Fig. 3a). Similar to Proteobacteria, *Fibrobacter* was increased with the extension of the retting time (Fig. 3a). At the genus level, *Prevotella* was the most dominant bacteria. The relative abundance of *Prevotella* at 0d and 10d was about 55%, but it decreased to 21.27% by the end of retting (Fig. 3b). In addition, the results showed that the relative abundance of *Acinetobacter* increased from 2.88% at the baseline to 6.64% at the 34d of retting (Fig. 3b). At the retting stage, some members of *Clostridium* were reduced from 3% at baseline to 2% at the 34d of retting (Fig. 3b). However, there were no significant differences between groups in several other bacteria with an average abundance greater than 1 %.

### Analysis of the carbohydrate active enzymes

**Fig. 4:**
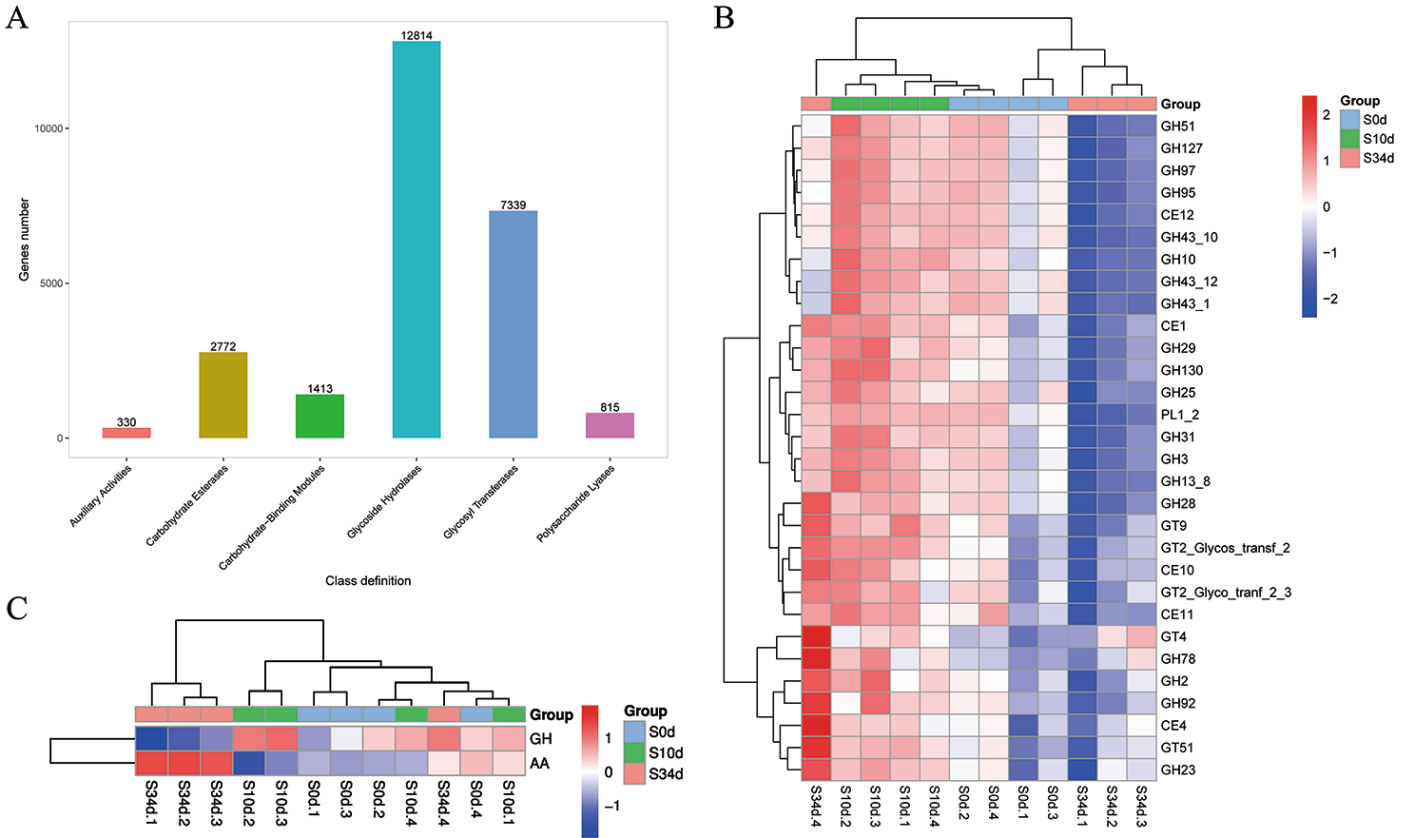
The carbohydrate active enzymes (CAZy) analysis results (a) Annotation of carbohydrate-related genes in the CAZy database. (b) The Cazy classification at the family level. Horizontal is sample information; Vertical is function annotation information; Red indicates high functional relative abundance, and blue indicates low functional relative abundance. (c) Functional classification with significant difference among groups with p value < 0.05. Vertical is function annotation information; Red indicates high functional relative abundance, and blue indicates low functional relative abundance. “S0d to S34d” refers to the type of samples (0d = baseline; 10 = retting samples for 10 days; 34d = retting samples for 34 days), the followed numbers refer to replicate samples

To identify the carbohydrate-active enzymes in the retting liquid, a similarity search of the identified proteins from the microbial community against the entire non-redundant sequences of the CAZy database was performed (Fig. 4a and b). This analysis demonstrated top 30 proteins assigned as putative CAZymes within the kenaf retting community. Overall distribution of proteins according to their CAZy families showed a large variety of functions.

On the other hand, Kruskal-Wallis data showed downregulation of the glycoside hydrolase families (GHs) in the 34d (Fig. 4c), while the Auxiliary Activities (AAs) were up-regulated in the 34d. Benzoquinone reductase, cellobiose dehydrogenase, glucose 1-oxidase, aryl alcohol oxidase and alcohol oxidase were the most abundant enzymes in the retting liquids. These observations demonstrated that there is constant change of rate of carbohydrate utilization with progression of retting.

### Proteomics data

**Fig. 5:**
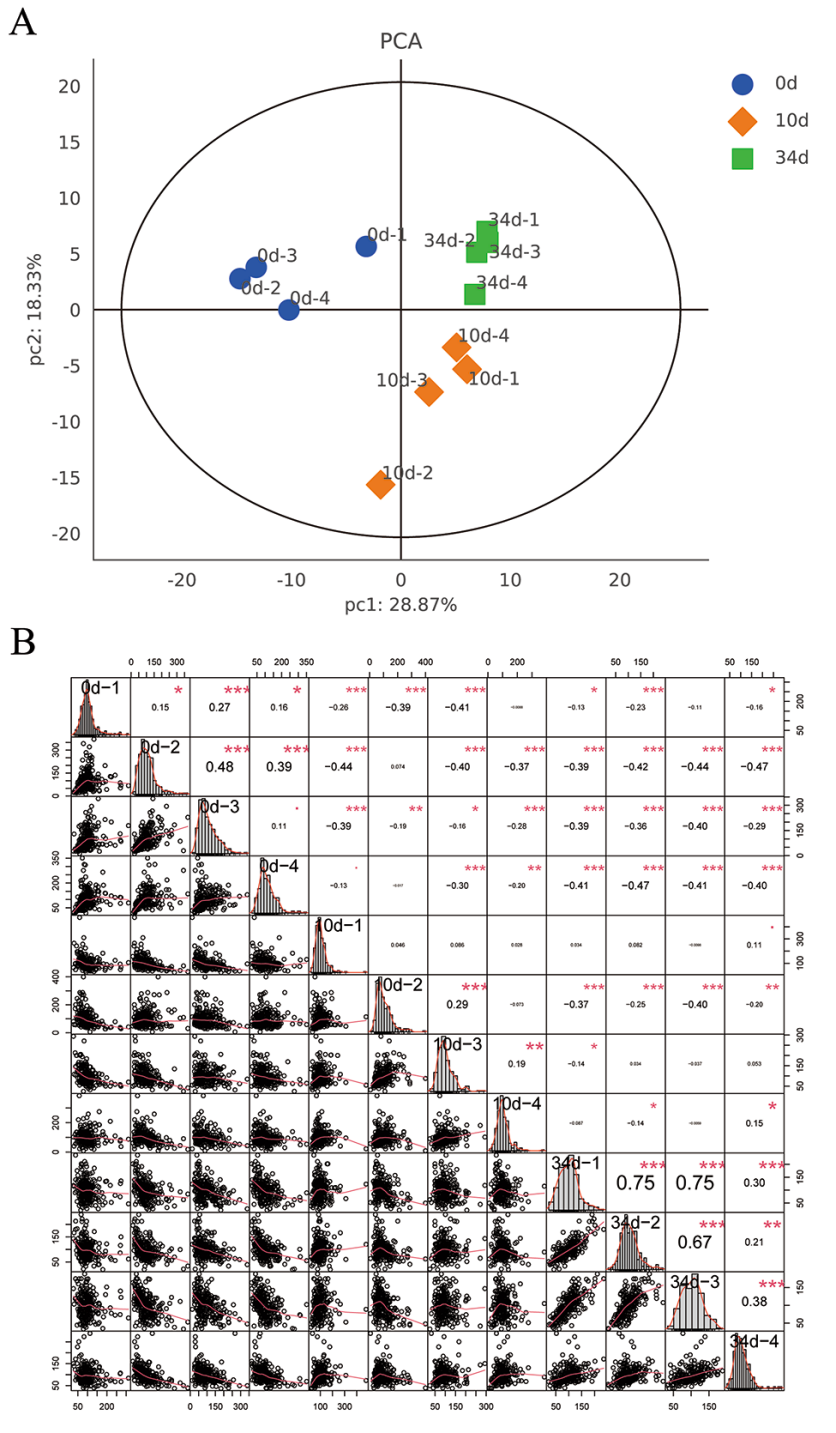
Proteomics data summary (a) Principal component analysis (PCA) score plot of proteomic data. (b) On the upper right of the diagonal line of the image, the number indicates the correlation value of the two samples, * indicates the degree of significance (* p < 0.05, ** p < 0.01, *** p < 0.001); on the lower left of the diagonal line, the scatter plot of the expression values of the two samples is shown. The red curve is the fitting trend. The larger the slope, the stronger the correlation between the two samples; the diagonal line is the distribution graph of the sample’s own expression level. “S0d to S34d” refers to the type of samples (0d = baseline; 10 = retting samples for 10 days; 34d = retting samples for 34 days), the followed numbers refer to replicate samples

The protein extracts prepared from the retting liquid samples were analyzed using LC-MS. Overall, 258 proteins and 314 peptides were detected in all the samples. Furthermore, PCA was used to evaluate the relationship among the biological replicates. The PCA results showed that samples in 0d were all located in the second quadrant; the samples in 10d were in the fourth quadrant (except for one in the third quadrant), while the samples in 34d were all located in the first quadrant. The PCA showed 28.87% (PC1) and 18.33% (PC2) of total variance (Fig. 5a). The sample-to-sample correlation analysis showed that samples in the same group had high correlation (Fig. 5b). The data demonstrated that the sample repeatability in the same group was good.

### Differentially expressed proteins and functional analysis

**Fig. 6:**
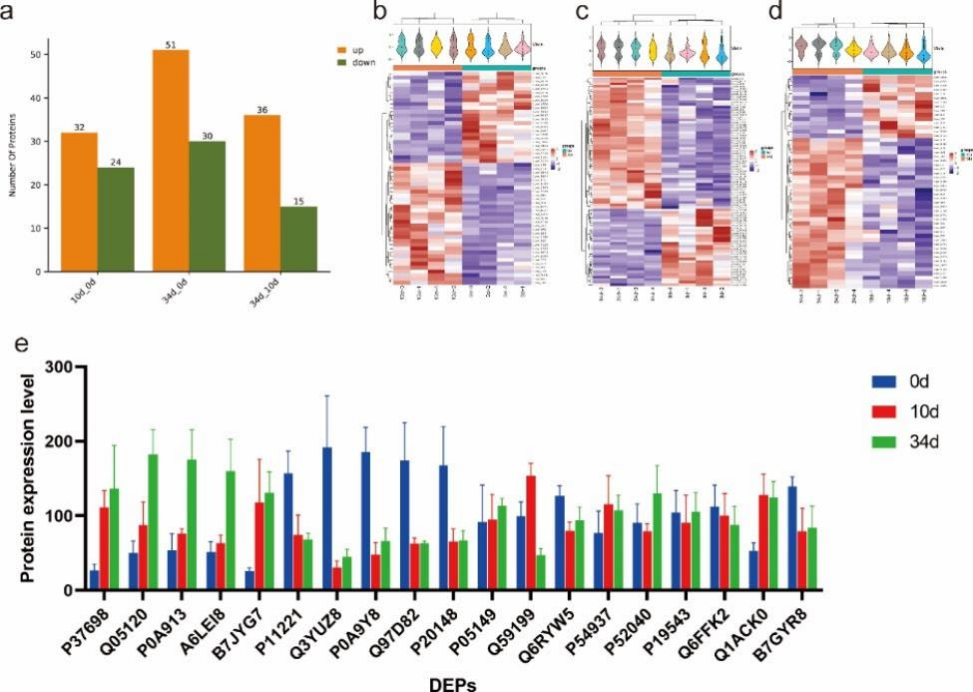
Summary of proteomic data and the expression of important differentially expressed proteins (DEPs) (a) The number of DEPs between different groups. (b-d) showed the heatmap of DEGs in 10d vs. 0d, 34d vs. 0d and 34d vs. 10d, respectively. The horizontal direction is the sample information; the vertical direction is the function annotation information; orange means the relative abundance of the function is high, and blue means the relative abundance of the function is low. (e) The expression of key DEPs in the three groups. “S0d to S34d” refers to the type of samples (0d = baseline; 10 = retting samples for 10 days; 34d = retting samples for 34 days), the followed numbers refer to replicate samples

This present study used proteomics to analyze differentially expressed proteins (DEPs) and their changes in the retting liquid. A total of 117 DEPs were identified in all the three groups (Table S2). Comparison of 10d vs. 0d, there were 32 and 24 DEPs were high and low expressed in 10d, respectively; for 34d vs. 0d, there were 51 upregulated and 30 down-regulated DEPs were identified; while in 34d vs. 10d, 36 and 15 DEPs were up-regulated and down-regulated, respectively (Fig. 6a). In addition, a heatmap was drawn to profile the expression of the DEPs in each comparison (Fig. 6b). Further analysis showed of the DEPs according to the protein annotation information identified the top five up-regulated (P37698, Q05120, P0A913, A6LEI8 and B7JYG7) and the top five down-regulated DEPs (P11221, Q3YUZ8, P0A9Y8, Q97D82 and P20148) with the largest fold change (Fig. 6c). The results showed that some of the DEPs were sharply changed at 10d, while others were changed at the 34d.

Further, Annotation information of all DEPs showed that P82593 is an important hemicellulose. KEGG enrichment analysis based on the DEPs identified in 10d vs. 0d and 34d vs. 0d was carried out. The results showed enrichment of five carbohydrate metabolism related pathways. Besides, three DEPs; P19543, Q1ACK0 and Q59199 were identified as the key indicators and the abundance of these three DEPs are as shown in Fig. 6d. P19543 is an oxidoreductase that is required for the transfer of electrons from pyruvate to flavodoxin; Q1ACK0 can improve oxidative fragmentation of the pentose substrate; while Gap play a role in catalyzing oxidative phosphorylation of glyceraldehyde 3-phosphate (G3P) to 1,3-bisphosphoglycerate (BPG) using the cofactor NAD. In addition, the data showed that gdhA (P95544), an important DEP in *Prevotella*, was enriched in pathways such as Nitrogen metabolism (ko00910), Alanine, aspartate, and glutamate metabolism (ko00250) and Arginine biosynthesis (ko00220).

In addition, we analyzed the expression of proteins in *Acinetobacter* and *Clostridium*. The protein expression profile in *Acinetobacter* showed that the average level of B7GYR8, Q6RYW5 and Q6FFK2 were reduced with the retting time, while P05149 was upregulated (Fig. 6e). For proteins in *Clostridium*, three degumming related proteins P37698, P52040 and P54937 were upregulated with the retting time (Fig. 6e).

### Redundancy analysis (RDA)

**Fig. 7:**
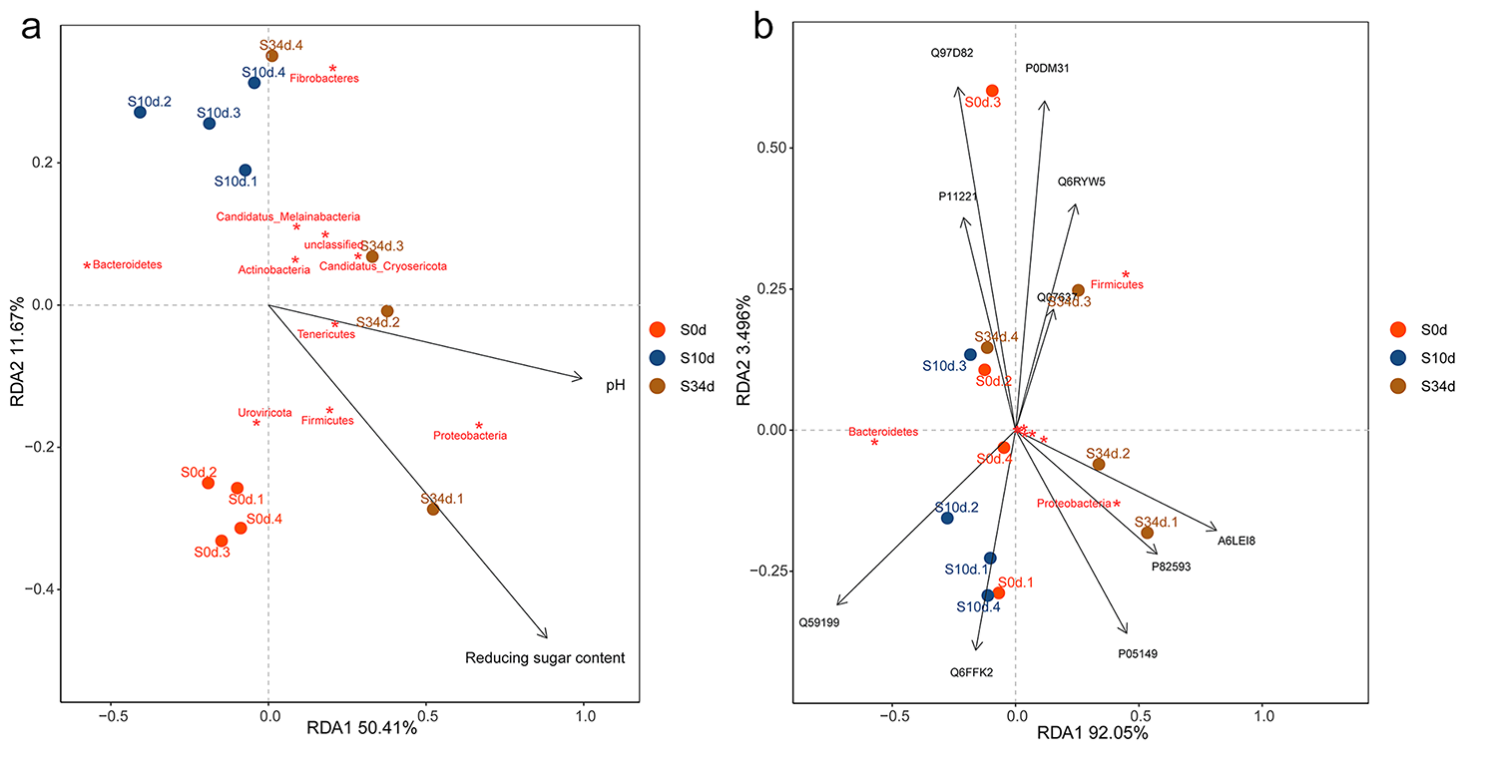
Redundancy analysis (RDA) ordination diagram. (a) RDA results between the top 20 phyla and environmental factors pH and Reducing sugar content. (b) RDA results between the top 20 phyla and the top 20 DEPs. “S0d to S34d” refers to the type of samples (0d = baseline; 10 = retting samples for 10 days; 34d = retting samples for 34 days), the followed numbers refer to replicate samples

RDA was performed using Omicshare (https://www.omicshare.com/) further highlighted these results. Firstly, the relationship between the top 20 phyla and environmental factors pH and reducing sugar content. The results showed that Tenericutes, Proteobacteria and Firmicutes were positively related to pH and reducing sugar content. Bacteroidetes were negatively connected with pH (Fig. 7 A). In addition, the top 20 phyla and the candidate DEPs that mentioned above were analyzed using RDA. The results showed that Firmicutes was positively related with Q07637, Q6RYW5, P0DM31, Q97D82 and P11221, but negatively with Q6FFK2. Proteobacteria was positively connected with P82593 (Fig. 7B).

## Discussion

Kenaf retting is a typical biochemical cycle of “gum nourishing communities, communities producing enzyme and enzyme degumming” [10]. In the early stage of degumming, microorganisms nourished water-soluble organic matter in kenaf to reproduction, resulting in significantly reduced sugar content and pH reduced from 6.6 to 6.4, which is a stage of “gum nourishing communities”. In the middle stage of degumming, microorganisms proliferate rapidly. Then, non-cellulosic degradation enzymes were secreted by the induction of kenaf gum in the successive stage, which belongs to the “communities producing enzyme”. In the later stage of degumming, non-cellulolytic enzymes will hydrolyze the kenaf gum and disperse kenaf fibers, which is the process of “enzyme degumming”. At the same time, abundant small molecules such as oligosaccharides or monosaccharides produced by this stage can be reused for the next cycle of microbial growth. However, with insufficient nutrients or excess metabolites in the retting pond, the growth and reproduction of microorganisms slowed down or autolyzed, which may lead to the pH of the kenaf-retting liquid rise to 7.3 [11].

Degumming of kenaf bast is a process mediated by dynamic changes of microbes. In this study, the growth of *Prevotella* was inhibited during the degumming of kenaf bast. Previous studies demonstrated that members of this genus produce pectinases and were identified as members of the ruminant gut [12–14]. Cheng showed that high molecular weight DNA with feruloyl esterase and xylanase activity might originate from *Prevotella* sp., and demonstrated its role in the degradation of straw [15]. Indeed, members of the dominant bacterium *Prevotella* were never associated with microbial retting process [6]. The proteomic and metagenomic analysis results do not support observations that *Prevotella* sp. could produce pectinase during the degumming process of kenaf. The significant reduction in the relative abundance of *Prevotella* sp. might be caused by the rising pH and competition for survival from other bacteria.

In addition, the previous data showed the lignin degrading role of *Acinetobacter* strains in hemp, ramie and mechanical pulp [16, 17]. For instance, Hu et al. observed that there was increased abundance of *Acinetobacter* post retting [18]. Some members, such as *Acinetobacter junii*, can be utilized in industrial enzymatic retting production once the process is optimized, and can replace the conventional chemical retting process due to its improved fiber quality and reduced environmental pollution [19]. In particular, the findings on *Acinetobacter* were consistent with this present result, which was increased from 2.88% at the baseline to 6.64% at the 34d of retting. The dynamic changes of *Acinetobacter* during the degumming of kenaf bast suggested its crucial roles in degrading kenaf. In addition, the results found that AAs were upregulated with the retting process, especially at 34d. Besides, the Kenaf fibers were the cleanest and smoothest at 34d. After the CAZy analysis, we demonstrated that cellobiose dehydrogenase and glucose 1-oxidase were more abundant in 34d compared to other time points. Although lignin breakdown enzymes may not act on carbohydrates, their association with carbohydrates in the plant cell wall makes the lignolytic enzymes cooperate with classical polysaccharide depolymerases [20]. We speculated that, cellobiose dehydrogenase and glucose 1-oxidase were in synergy with pectinase, and the action of cellobiose dehydrogenase and glucose 1-oxidase may be key in keeping the kenaf fiber smooth.

From the structure of kenaf, its pectin mainly composed of D-galacturonic acid ɑ- A polysaccharide chain, which was formed by polymerization of 1,4 glycosidic bonds [21]. Therefore, glycosidase may break the polysaccharide chain connecting pectin during the degumming process. Hemicellulose is mainly composed of polyacetal, polyhexose and polypentose, and aldehyde dehydrogenase can act on hemicellulose in conjunction with glycosidase [22]. On the other hand, the proteomics data showed that many DEPs from bacteria. Detailed analysis of the DEPs showed that P37698 play a role in the degradation of cellulose or related beta-glucans. Furthermore, P37698 was derived from *Clostridium*. Duan reported that *Clostridium* produced pectinase and mannanase in kenaf retting process, and the existence of *Clostridium* could shorten the degumming time [7]. The upregulation of P37698, P52040 and P54937 (not statistically different) in *Clostridium* indicated that the decomposition reaction of cellulose and pectin increased with the extension of the retting time. Together, this present result showed that *Clostridium* is a critical bacterium that helps degumming. The upregulation of P37698, P52040 and P54937 proteins are an important manifestation and plays an important role in the degumming process. Although the abundance of *Clostridium* demonstrates that it is not the dominant bacteria, a small amount of *Clostridium* mediates important roles. RDA results showed the positive and relationship between *Firmicutes* and six DEPs (Q07637, Q6RYW5, P0DM31, Q97D82 and P11221), respectively. Many *Firmicutes* produce endospores, which are resistant to desiccation and can survive extreme conditions. *Bacilli* class is the most abundant bacteria of *Firmicutes* phylum. As we know, *Bacilli* is an important degumming bacterium [23–27]. Previous studies found that *Bacillus* could produce pectinase [28, 29]. Except pectinase, some *Bacillus* have the ability to produce specific degumming enzymes, including cellulase and xylanase [30]. However, no pectinase was detected in this present study, which might be caused by other major isoenzymes. But more work is needed to prove this conjecture. In addition, we found that *Proteobacteria* was positively connected with P82593. According to the annotation information, P82593 is extracellular exo-alpha-L-arabinofuranosidase. As we know, various plant cell wall polymers contain arabinose, which is the second most abundant pentose in nature [31]. Arabinose-containing polysaccharides such as arabinoxylan (AX), arabinan and arabinogalactan (AG) are found as major components of hemicelluloses and pectic substances, respectively [32]. Arabinans and arabinogalactans are constituents of pectic substances [33]. Alpha-L-arabinofuranosidases are involved in the hydrolysis of multiple alpha-L-arabinosyl linkages in oligosaccharides, and in hemicelluloses distributed in various plant tissues such as arabinoxylans, arabinogalactans and arabinans [34]. These enzymes work in synergy with other hemicellulolytic enzymes to achieve the complete degradation of the hemicelluloses. In this present study, the content of P82593 was increased with the extension of the retting time, especially after 10 days. Previous studies reported that most α-L-arabinofuranosidases are optimally active under neutral pH (pH 6.0–7.0) and mesophilic (30–50 °C) conditions [35]. In this study, the pH and temperature of the retting environment were close to the optimal conditions of α-L-arabinofuranosidases after 20 days, so we believe that the last two weeks of retting are the fastest and most critical time of the whole retting process, and this is also consistent with the observation of scanning electron microscope. Some bacteria in *Bacillus* class can produce α-L-arabinofuranosidases [36, 37]. Therefore, bacteria *Bacillus* and protein P82593 plays an important role in the kenaf bast degumming process.

## Conclusion

Taken together, this present study demonstrated that growth of *Prevotella* was initially inhibited with the kenaf retting. The abundance of *Acinetobacter* and *Bacilli* class (the most abundant bacteria of *Firmicutes* phylum) were increased during the degumming process, while *Clostridium* maintained a constant level. Secretory proteomics analysis showed that the upregulation of P37698, P52040, P54937 and P82593 proteins is an important phenotype and played an important role in the degumming process. In particular, P82593, as a hemicellulase, may play a decisive role in the degumming process of kenaf bast.

## Materials and methods

### Retting system establishment

**Fig. 8:**
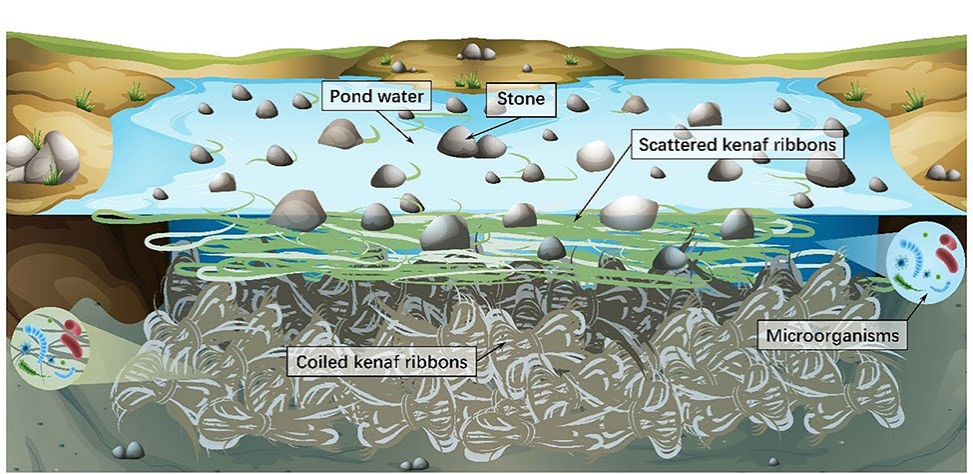
Construction of kenaf retting system.The bottom layer of brown-yellow filamentous band is the coiled kenaf bast, the middle green ribbon is scattered kenaf bast, and the block above is stone. With scattered kenaf bast and stones press on coiled kenaf bast, so that coiled kenaf bast is completely submerged in the pool water for up to 34 days of retting, and kenaf retting liquid was collected for sample at 0 days, 10 days, 20 days and 34 days respectively

The retting pond was dug on site in kenaf planting field, and the volume of the pond was about 6 (length)×8 (width)×1.4 (depth) m^3^; the water was injected into the pond, and the volume of the water was about 75% of the volume of the pond; 0.5% (w/v) urea was added as a degumming aid. Putting the peeled kenaf ribbons into the retting pond, lay them flat. Then laying the scattered kenaf on top, then grasses on top of the kenaf, finally compact it with soil and stone (Fig. 8).

### Plant materials and sampling

The common kenaf variety, Zhong-za-hong-318, was used in this study. Seeds were obtained from the National Bast Germplasm Resources Medium-term Bank in Institute of Bast Fiber Crops, Chinese Academy of Agricultural Science and then sown on May 31, 2020. The crops were harvested on September 30, 2020, with a growth period 122 days. Kenaf planting conditions were undertaken following the conventional planting methods. After harvesting of the kenaf, we used 4HB-480 jute kenaf peeling machine to separate the kenaf bark and stem. Water retting was performed at local fiber processing sites where the duration, type of water, type of container, pre-treatment process, and drying period were executed according to our previous report.

Retting liquid samples were collected on the 0d, 10d, 20d, 30d and 34d of the retting process. For each liquid sampling, 4 sampling sites in the retting pool (sampling 3ml of liquid at 10 cm below the liquid surface each time) were randomly used as biological repeats. The liquid samples were quickly frozen in liquid nitrogen and then stored at -80℃.

For kenaf fiber sampling, five samples (biological replicates) were randomly selected from the retting pool, at a sampling time like that used for liquid samples. Thereafter, the fiber samples were divided into two parts, one was stored in liquid nitrogen, and the other part was cleaned and dried for electron microscopy scanning.

### Determination of physical and chemical characteristics of retting liquid

To evaluate the physical and chemical properties of the retting liquid, the pH and reducing sugars of the liquid at different stages was measured in the retting process. The pH value was assessed using an automated pH analyzer (pH-Meter HI9025, Hanna Instruments, Vöhringen, Germany), while the reducing sugar was determined using the DNS method [38]. Data were computed into the standard curve to estimate the content of the reducing sugar of the retting liquid.

### Determination of fiber yield

The fiber yield was calculated using the formula:

1 $$Fiber{\text{ }}yields{\text{ }} = \left( {\frac{{{G_f}}}{{{G_m}}}} \right) \times 100$$

, where ${G_m}$ is the quality of dried kenaf raw material and ${G_f}$ is the quality of dry kenaf fiber.

### Observation of fiber morphology

To evaluate the morphology and diameter of the fibers, the materials were gold-coated and observed under scanning electron microscope (SEM, SH-5000 M, HIROX, Japan) at an accelerating voltage of 15 kV. For each sample (n = 3), five random spots were captured to generate micrographs.

### Next-generation sequencing of the retting liquid microbiome

Metagenomic sequencing of the retting liquid microbiome was performed using next-generation sequencing. Bacterial DNA was extracted from the retting liquid samples at different times using a Power Water DNA Isolation Kit (Qiagen, Valencia, CA, USA) according to the manufacturer’s instructions. DNA library preparation for metagenome sequencing was performed was conducted by OE biotech Co., Ltd. (Shanghai, China). Libraries were sequenced using Illumina MiSeq following Truseq DNA library preparation protocol, and then the sequence files were processed as previously described [39]. The raw data was stored in FASTQ format and the Reads were trimmed and then filtered using Trimmomatic (v0.36). After obtaining valid reads, metagenome assembly was performed using MEGAHIT (v1.1.2). Gaps inside scaffolds were used as breakpoints to interrupt the scaffold into new contigs. ORF prediction of the assembled scaffolds was performed and translated into amino acid sequences using prodigal (v2.6.3). The gene set representative sequence (amino acid sequence) was annotated with NR, KEGG, COG, SWISSPROT and GO database, with an e-value of 1e-5. The taxonomy of the species was obtained with reference from the NR Library, while the abundance of the species was calculated using the corresponding abundance of the genes. To profile the abundance on the corresponding taxonomics level, abundance statistics were performed at each level of Domain, Kingdom, Phylum, Class, Order, Family, Genus, Species.

### Proteome sample preparation, TMT labeling and LC–MS/MS analysis

The retting liquid samples were filtered and then collected. The protein concentration was determined using EZQ protein quantitation method following the manufacturer’s instructions (Bio-Rad, Hercules, CA, USA). For TMT labelling, the lyophilized samples were resuspended in 100 µL of 200 mM TEAB, and then 40 µL of each sample were transferred into new tubes for labeling. Eighty-eight microliters of acetonitrile were added to the TMT reagent vial at room temperature. The reagents were dissolved for 5 min, mixed and then centrifuged. Then 41 µL of the TMT label reagent was added to each sample and the tubes were incubated at room temperature for 1 h. To terminate the reaction, 8 µL of 5% hydroxylamine were added to each sample and incubated for 15 min. Digested peptide samples were analyzed using LC–MS/MS at the Shanghai Luming Biotech. Co., Ltd (Luming, Shanghai, China). The peptides were then separated and analyzed using a Michrom Paradigm Multi-Dimensional Liquid Chromatography instrument (Michrom Bioresources Inc., Auburn, CA, USA) coupled with a Thermo LTQ Orbitrap XL mass spectrometer (Thermo Fisher Scientific, San Jose, CA, USA). The peptide samples were dissolved in 100 µL of 0.1% formic acid and loaded onto a ZORBAX 300SB-C18 5-µm (5 × 0.3 mm) trap column (Agilent Technologies, Santa Clara, CA, USA) before elution. Thereafter, the samples were separated with a reverse phase Michrom Magic C18AQ column (3 μm, 200 Å, 0.2 × 150 mm) through gradient elution using solvent A (0.1% formic acid) and solvent B (0.1% formic acid in ACN), at a flow rate of 2 µL min − 1. The gradient was set from 5 to 40% solvent B for 90 min, increased to 80% solvent B in 10 s and then held at 80% solvent B for 1 min. The MS spectra were recorded over the mass range of m/z 400–1600 with a resolution of 60 000. The three most intense ions were isolated for fragmentation in the linear ion trap using CID with a minimal signal of 500 and collision energy of 35.0 or using HCD with a minimal signal of 1000, collision energy of 55.0, and an activation time of 30 ms. Dynamic exclusion was implemented with two repeat counts, a repeat duration of 15 s and exclusion duration of 90 s.

### Processing of proteome data

The MS/MS data were imported in Maxquant to label free quantification analysis and using Andromeda engine search engine. This search engine was used to analyze the UniProt Citrus sinensis database assuming digestion with trypsin. The Andromeda search was performed with a fragment ion mass tolerance of 0.020 Da and a parent ion tolerance of 5.0 ppm. In addition, Scaffold Q+ (version Scaffold_4.6.2, Proteome Software Inc., Portland, OR, USA) was used to quantify TMT Label Based Quantitation peptide and protein identification. Normalization was performed iteratively (across samples and spectra) on intensities, as described in Statistical Analysis of Relative Labeled Mass Spectrometry Data from Complex Samples Using ANOVA. Besides, medians were used for averaging. Spectra data were log-transformed, pruned of those matched to multiple proteins and weighted using an adaptive intensity weighting algorithm. We determined differentially expressed proteins using a Mann-Whitney test with unadjusted significance level p < 0.05 corrected by Benjamini-Hochberg.

### Statistical analysis

The fiber yields data were presented as a mean ± SD. Statistical analysis was conducted by the Student’s t-test or ANOVA analysis. A p-value < 0.05 was considered statistically significant.

## Electronic supplementary material

Below is the link to the electronic supplementary material.

Supplementary Material 1

Supplementary Material 2

## Data Availability

Metagenomic sequencing data files have been uploaded to the Sequencing Read Archive (PRJNA850647). All proteome raw data were uploaded to the ProteomeXchange Consortium (proteomecentral.proteomexchange.org) with the access ID of PXD035056.

## Declarations

### Rights and licenses

This study was approved by the Institute of Bast Fiber Crops of Chinese Academy of Agricultural Science, and the kenaf seeds were obtained from the seed resource bank of the Institute.
